# Correction: Satam et al. Next-Generation Sequencing Technology: Current Trends and Advancements. *Biology* 2023, *12*, 997

**DOI:** 10.3390/biology13050286

**Published:** 2024-04-24

**Authors:** Heena Satam, Kandarp Joshi, Upasana Mangrolia, Sanober Waghoo, Gulnaz Zaidi, Shravani Rawool, Ritesh P. Thakare, Shahid Banday, Alok K. Mishra, Gautam Das, Sunil K. Malonia

**Affiliations:** 1 miBiome Therapeutics, Mumbai 400102, India; 2 Department of Molecular Cell and Cancer Biology, UMass Chan Medical School, Worcester, MA 01605, USAalok.mishra@umassmed.edu (A.K.M.)

We are very thankful to the commentator for pointing out the issues in the review article by Satam et al. [1]. As noted by the commentator, there were a few discrepancies related to the information about the PacBio system in the review article by Satam et al., as discussed below.

The commentator highlighted the point in the original article regarding the higher error rate of long-read sequencing compared to short-read sequencing. We presented this as a general statement for long-read sequencing technologies, without specifically mentioning PacBio. We acknowledge the updated information and the references cited by the commentator. After considering the references cited by the commentator and the study from the Association of Biomolecular Resource Facilities (ABRF), we agree with the updated information provided by the commentator that PacBio CCS has the lowest error rate among all sequencing technologies.

The commentator identified an error in Figure 2 and Table 1 of the original article. We appreciate the raised comment and acknowledge that it was an unintentional typographical mistake. In the subsequent paragraph of the original article, where we compare short-read and long-read sequencing, we used the term ‘PCR-Free’. We apologize for the typographical error in Figure 2 of the article, which has now been rectified (see the corrected figure below). Furthermore, we have also corrected this information in Table 1 (see below).

**Figure 2:**
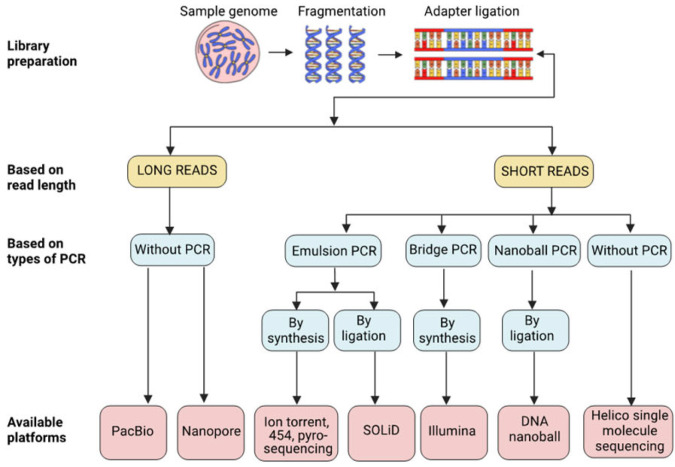
Overview of various NGS technologies with different platforms and principles.

**Table 1:** Different generations of NGS platforms.

Sr No.	Platform	Use	Sequencing Technology	Amplification Type	Principle	Read Length (bp)	Limitations	Ref.
1	454 pyrosequencing	Short read sequencing	Seq by synthesis	Emulsion PCR	Detection of pyrophosphate released during nucleotide incorporation.	400–1000	May contain deletion and insertion sequencing errors due to inefficient determination of homopolymer length.	[18–20]
2	Ion Torrent	Short read sequencing	Seq by synthesis	Emulsion PCR	Ion semiconductor sequencing principle detecting H^+^ ion generated during nucleotide incorporation.	200–400	When homopolymer sequences are sequenced, it may lead to loss in signal strength.	[19–21]
3	Illumina	Short read sequencing	Seq by synthesis	Bridge PCR	Solid-phase sequencing on immobilized surface leveraging clonal array formation using proprietary reversible terminator technology for rapid and accurate large-scale sequencing using single labeled dNTPs, which is added to the nucleic acid chain.	36–300	In case of sample overloading, the sequencing may result in overcrowding or overlapping signals, thus spiking the error rate up to 1%.	[19,20,22]
4	SOLiD	Short read sequencing	Seq by ligation	Emulsion PCR	An enzymatic method of sequencing using DNA ligase. 8-Mer probes with a hydroxyl group at 3′ end and a fluorescent tag (unique to each base A, T, G, C) at 5′ end are used in ligation reaction.	75	This platform displays substitution errors and may also under-represent GC-rich regions. Their short reads also limit their wider applications.	[20,23]
5	DNA nanoball sequencing	Short read sequencing	Seq by ligation	Amplification by Nanoball PCR	Splint oligo hybridization with post-PCR amplicon from libraries helps in the formation of circles. This circular ssDNA acts as the DNA template to generate a long string of DNA that self-assembles into a tight DNA nanoball. These are added to the aminosilane (positively charged)-coated flow cell to allow patterned binding of the DNA nanoballs. The fluorescently tagged bases are incorporated into the DNA strand, and the release of the fluorescent tag is captured using imaging techniques.	50–150	Multiple PCR cycles are needed with a more exhaustive workflow. This, combined with the output of short-read sequencing, can be a possible limitation.	[24,25]
6	Helicos single-molecule sequencing	Short-read sequencing	Seq by synthesis	Without Amplification	Poly-A-tailed short 100–200 bp fragmented genomic DNA is sequenced on poly-T oligo-coated flow cells using fluorescently labeled 4 dNTPS. The signal released upon adding each nucleotide is captured.	35	Highly sensitive instrumentation required. As the sequence length increases, the percentage of strands that can be utilized decreases.	[26,27]
7	PacBio Onso system	Short-read sequencing	Seq by binding	Optional PCR	Sequencing by binding (SBB) chemistry uses native nucleotides and scarless incorporation under optimized conditions for binding and extension (https://www.pacb.com/technology/sequencing-by-binding/, accessed on 1 July 2023).	100–200	The higher cost compared to other sequencing platforms.	
8	PacBio Single-molecule real-time sequencing (SMRT) technology	Long-read sequencing	Seq by synthesis	Without PCR	The SMRT sequencing employs SMRT Cell, housing numerous small wells known as zero-mode waveguides (ZMWs). Individual DNA molecules are immobilized within these wells, emitting light as the polymerase incorporates each nucleotide, allowing real-time measurement of nucleotide incorporation	average 10,000–25,000	The higher cost compared to other sequencing platforms.	[28,29]
9	Nanopore DNA sequencing	Long-read sequencing	Sequence detection through electrical impedance	Without PCR	The method relies on the linearization of DNA or RNA molecules and their capability to move through a biological pore called “nanopores”, which are eight nanometers wide. Electrophoretic mobility allows the passage of linear nucleic acid strand, which in turn is capable of generating a current signal.	average 10,000–30,000	The error rate can spike up to 15%, especially with low-complexity sequences. Compared to short-read sequencers, it has a lower read accuracy.	[5,19,30]

The commentator drew attention to the issue regarding the read length of PacBio as stated in the original article. We originally mentioned a read length of 10–16 kb. We acknowledge that the reference we cited was old, and the references cited by the commentator for this are the most recent and updated, suggesting a read length of 15–25 kb. Therefore, we revised our statement accordingly that the average read length of the PacBio system is 10–25 kb (See corrected Table 1 above).

The commentator highlighted statements related to PacBio SMRT sequencing, including ‘in wells where high processive DNA is prebound’, ‘fluorescently labeled nucleotides which upon incorporation emit a fluorescent signal’, and ‘the molecule quickly diffuses’ (Table 1). We acknowledge that these statements were not correctly phrased. The information was sourced from a review article by Mantere et al. which describes these details using similar terms, such as ‘pre-bound polymerase’ and ‘incorporation of the labeled bases,’ in the technical summary of SMRT in their review article. In light of the raised comments and for better clarity for readers, we have revised these statements (See Table 1)

In the context of the comment on the low throughput of the PacBio SMRT system, we accept that appropriate references were not quoted; we apologize for that. In light of the comment and the reference cited by the commentator, we revise our statement that the PacBio Pac Bio SMRT system has a high throughput.

The commentator has pointed out that our statement regarding PacBio SMRT sequencing having ‘low flow cell success’ (Table 1 of the original article) is inaccurate. He clarifies that PacBio SMRT sequencing does not utilize flow cells, and there is no flow of any reagents during the sequencing reaction. We apologize if this information was not accurately presented in the original article’s table, and we want to clarify that we did not intend to damage PacBio’s reputation. After carefully evaluating comments and cited references, we have revised these statements in the table (see above Table 1).

The commentator highlighted the statements on PacBio Onso short-read sequencing. We initially mentioned, “The minimum data are 80 GB with 200 cycles, which necessitates a higher sample requirement (Table 1). After careful examination of the facts and comments, we agree that neither the minimum amount of data nor the number of cycles dictate sample input requirements, as SBB chemistry can utilize PCR amplification just like other short-read technologies. The authors accept the comment made on the relation between the sample input requirement and data output. We agree with the fact that Onso systems work with as low as 10 ng input.

We thank the commentator for raising this issue and providing us with the most relevant information that helped us convey the most updated information in the related field.

The authors state that the scientific conclusions are unaffected. This correction was approved by the Academic Editor. The original publication has also been updated.
